# Focusing Treatment on Pregnant Women With COVID Disease

**DOI:** 10.3389/fgwh.2021.590945

**Published:** 2021-10-11

**Authors:** Alina-Raluca Emanoil, Emanuela Stochino Loi, Anis Feki, Nordine Ben Ali

**Affiliations:** Department of Gynecology and Obstetrics, Fribourg Cantonal Hospital, Fribourg, Switzerland

**Keywords:** COVID-19, SARS-CoV-2, pregnancy, coronavirus, treatment, COVID-19 vaccine

## Abstract

Since the emergence of a novel coronavirus in China at the end of December 2019, its infection - COVID-19 - has been associated with high morbidity and mortality and has left healthcare systems wrestling with the optimal management strategy, especially for vulnerable populations, such as pregnant women. At this moment, few resources exist to guide the multi-disciplinary team through decisions regarding optimal maternal-fetal treatment and delivery timing. In this article, we present the drugs and vaccines under investigation as potential treatments and prevention for COVID-19 infection. Based on a comprehensive evaluation, we prioritized these possible treatments, and provide dose-response and dose-toxicity information on each drug. Currently, there is limited but very increasing reassuring information concerning vaccines to prevent SARS-CoV-2 during pregnancy, and in this review, we also emphasize the results (mostly positive) provided by the few small trials evaluating COVID-19 vaccines in pregnant patients.

## Introduction

Coronavirus 2019 disease (COVID-19) is a recently emerged infection caused by a ribonucleic acid virus that leads to mild to severe respiratory tract infections (1). This virus emerged in December 2019 in China and was named severe acute respiratory syndrome-related coronavirus-2 (SARS-CoV-2) (2, 3). The main source of infection is people already infected with SARS-CoV-2. Asymptomatic carriers can also be a source of infection. The transmission routes are via airborne exposure, via droplets and close contact (4). According to the World Health Organization (WHO), international statistics have demonstrated the severity of this public health crisis and that COVID-19 infected pregnant women are a potentially vulnerable population (5–8).

In this review, we assessed the potential strategies for optimal maternal treatment, fetal surveillance and delivery timing, taking into account that pregnant women have a modified immune and respiratory system, especially at the end of the gestation period, making them more susceptible to severe symptoms such as pneumonia and marked hypoxia (9, 10). At the same time, we reviewed the subject of SARS-CoV-2 vaccines safety and efficacy for pregnant and lactating women. The latest publications on this subject recommend to consider COVID-19 vaccination during pregnancy, especially for the pregnant women who present a higher risk of exposure or severe disease if infected. For this vulnerable population, it would be better to inject the COVID-19 vaccine during pregnancy than to postpone vaccination until the postpartum period. Also, it is important to acknowledge the timing of SARS-CoV-2 vaccination in pregnant women. They should not receive this type of vaccine within 14 days of the administration of a routine vaccine (e.g., influenza). As an exception, this interval may be shortened in the case of important vaccines in a life-threatening situation (e.g., tetanus vaccination following wound treatment) (11–21).

The WHO has identified four clinical stages of COVID-19 infection: *a mild form* in patients with no specific symptoms (fatigue, cough, muscle pain, nasal congestion, headache, fever, sore throat, sometimes nausea, diarrhea, vomiting and anosmia); a *moderate form* with pneumonia but no need for supplemental oxygen; a *severe form* of pneumonia with the need for oxygen and a *very severe form* requiring mechanical ventilation, sometimes accompanied by shock and organ failure (4). Comorbidities that usually appear in the second trimester of pregnancy (hypertension, cholestasis, diabetes), as well as obesity and increased maternal age, often make pregnant women more susceptible to severe COVID-19 symptoms, which will put them at a higher risk of being admitted to the intensive care unit with mechanical ventilation compared to the general population (22).

There is no clear evidence that the coronavirus has an impact of the fetus, either in the first or second trimester as miscarriage or late pregnancy loss, nor as preterm birth, whether this is iatrogenic (maternal viral infection) or spontaneous (prelabour premature rupture of the membranes) (7, 23–26). Many studies are in progress, and there is already a meta-analysis published suggesting that patients with severe COVID-19 symptoms during pregnancy may experience spontaneous premature birth and, as a consequence, their newborns require the neonatal department for vital support (27). On the other hand, vertical transmission is not excluded, even though there have been no studies showing the presence of SARS-CoV-2 in the placenta, amniotic fluid, cord blood, neonatal throat or breast milk (24–26, 28, 29). Mazur-Bialy et al. indicated a very slight possibility (about 3–8%) of vertical transmission from women infected with coronavirus to their newborns (22).

Due to the absence of clear guidelines based on conclusive studies, at this moment, there is an urgent need for effective treatment strategies for COVID-19, especially for pregnant women (30). With this purpose, a very good critical review by Favilli et al. was published on the effectiveness and safety of available treatments for COVID-19 during pregnancy, drawing attention to the potential adverse fetal and neonatal effects of drugs, as this is an important problem for medical practitioners (31). At the same time, more data concerning the safety and efficacy of SARS-CoV-2 vaccines during pregnancy and postpartum is essential for both obstetricians and patients (11). In this article we performed a literature review for relevant publications appearing in the scientific database up to 7 May, 2021.

## Prevention

There are currently no data suggesting clear management strategies for treating COVID-19 infection in pregnant woman, so prevention remains the principal strategy. There is evidence of two routes for human transmission: direct (contact with an infected person within 2 m) and indirect (by touching an object already touched by an infected person) (30). For these reasons, pregnant women should be advised to avoid close contact by maintaining the proper distance, wash their hands often with soap and water, disinfect touched surfaces daily, use a face mask in the community, avoid contact with vulnerable groups (people with cancer, people with known immunosuppression or organ transplant recipients), stay at home if sick and cove the mouth and nose when coughing or sneezing (32).

In the postpartum period, women with COVID-19 infection and willing to breastfeed should take precautions in order to protect the newborn: correct hand hygiene before and after contact, cleaning the breast skin and use of a face mask. According to the current evidence, international scientific organizations allow breastfeeding if both maternal and neonatal conditions are favorable (33, 34).

All around the world, when evaluating COVID-19 vaccines, trials have excluded pregnant women and those breastfeeding because of the limited data about safety and efficacy in this vulnerable population (11). So, considering that there is still no specific treatment for COVID infection and no specific COVID-19 vaccine for pregnant and lactating women, it should be well-acknowledged that prevention is the best option.

## Vaccines

There are numerous vaccines to prevent infection to SARS-CoV-2 available worldwide, the two basic principles being the mRNA vaccine and the replication-incompetent adenovirus recombinant vector vaccine. They do not contain virus that replicates and they do not cause disease, but there may be some non-specific side effects due to activation of the immune system (11).

The COVID-19 vaccines have been granted emergency use of authorization in specific doses and series for individuals older than 18 years of age. Usually, they are administered intramuscularly (into the deltoid) in a two-dose series or only one dose, depending on the type of vaccine (12, 20). The interval of time between the two doses is 21–28 days, specifically for each type of vaccine. If impossible to respect this timing, it is suggested to administer the second dose as soon as possible, but no longer than 42 days after the first dose (12, 19). Ideally, the type of vaccine initially used as first dose should be the same for the second dose (19) as there is no data to confirm the efficacy and safety if using two different vaccines for each dose (12).

Even though experts believe that these vaccines (both mRNA and viral vector) do not present a risk either for pregnant women or for their fetus or breastfeeding newborn (21), trials that have evaluated COVID-19 vaccines until now have excluded pregnant and lactating women. Therefore, current date is from animal studies and small prospective cohort studies on vaccinated pregnant women (11, 13). Researchers have demonstrated that antibody titres after maternal vaccination were higher than those induced by COVID-19 infection during pregnancy. Most importantly, they were able to identify vaccine-generated antibodies in umbilical cord blood and breastmilk samples (13).

Some reports on vaccination among pregnant women (mainly vaccinated in the third trimester) show no evidence of harmful effects such as neonatal death, stillbirth, congenital anomalies, fetal growth, preterm birth or miscarriage (11, 17). These are the arguments why experts strongly advise that pregnant patients have the COVID-19 vaccine, especially those who present an important risk of exposure to SARS-CoV-2 (e.g., health care workers) or with comorbidities (e.g., obesity, diabetes, heart disease) that will increase their risk of developing a severe disease if infected with coronavirus (11, 20)^1^.

It is well-known that pregnancy itself represents a high risk of severe infection, but due to the limited current data about the safety and efficacy of vaccines to prevent SARS-CoV-2 during the gestational period, some pregnant women may choose to defer COVID-19 vaccination, for the moment (11, 12). When women opt for COVID-19 vaccine during pregnancy the timing with non-COVID-19 vaccines should not be neglected. As there is no information about the safety and efficacy of SARS-CoV-2 vaccines being co-administered with other vaccines, an interval of 14 days has been suggested between COVID-19 vaccine and others. There may be some exceptions, such as the tetanus vaccination in case of injury and wound treatment (12).

One of the major side effects after undergoing COVID-19 vaccine is thrombosis. There are few cases mentioned in the literature at this moment (thrombosis associated with thrombocytopenia) and they are reported especially after viral vector vaccines (35). Taking into consideration this data, experts on the subject suggest that women during gestational or postpartum period should opt for mRNA vaccines if accessible. If not, they believe that any viral vector vaccine would be better than no vaccine at all (11). Another relevant aspect on this topic is that RhD alloimmunisation seems to have no interference with the immune response when pregnant patients choose to get vaccinated against SARS-CoV-2. So, the Anti-D immunoglobulin should be administered as standard clinical protocols recommend (11, 36). Concerning the timing of a pregnancy after undergoing the first or both COVID-19 vaccine series, experts believe that there is no impact on pregnancy and that vaccination against SARS-CoV-2 infection should take place or continue based on standard protocols (11). Another key matter related to the COVID-19 vaccines is the breastfeeding. As we specified in the beginning of this topic, lactating patients were excluded from vaccine trials. Officially, breastfeeding is not an exclusion criteria as specialists have demonstrated that patient COVID-19 antibodies after vaccination may have a potential protective effects on the newborn, by crossing into the breastmilk (11, 16, 37).

Nowadays, the published vaccine registries report no significant risk to either the pregnant woman or her fetus (11).

## Treatments

In the current literature, there is limited information on the effects of drugs in pregnant women affected by coronavirus. Clinical findings are similar in the case of non-pregnant adults, but knowing that the immune system changes during gestation, pregnant women might be at a greater risk for morbidity and mortality related to COVID-19 compared to the general population. Clearly, pregnant woman should receive the same care as other people regarding screening, radiology and laboratory evaluations as well as treatment and critic care (38).

As very clearly stated by Favilli et al. (31), COVID-19 treatment and especially antiviral drugs during pregnancy may be difficult to manage considering that it is part of their life cycle for the viruses to mutate constantly and so it is a challenge to develop curative drugs. Moreover, clinical trials do not include pregnant and lactating women and therefore, antiviral drugs that are safe and effective in general population cannot be used during pregnancy and breastfeeding (39).

The management of COVID-19 in pregnant women in terms of prescribing pharmacological treatment must take into consideration the gestational age in order to minimize fetal risks. In the severe forms of the disease, it is suggested to end the pregnancy by cesarean section before starting treatment, but always with the consent of the patient (4). The standard treatment for patients who need only home isolation includes bed rest, hydration, adequate calorie intake, paracetamol up to 4 g/day and antiviral drugs, but with continuous evaluation of the effectiveness of the drugs in use by routine visits (medical staff), at home preferably, at least four times per week (40).

There are still several drugs being used off-label, and it is important to note that there may be serious adverse effects. Below we will list the drugs that are available for the management of COVID in pregnant women and in the immediate postpartum period. We focused on the safety and effectiveness of currently known treatments for COVID-19 infection during pregnancy after analyzing clinical studies and literature reviews.

## Antimalarials

### Chloroquine and Hydroxychloroquine

Chloroquine and hydroxychloroquine (HCQ) are oral drugs are used for the treatment of malaria and some autoimmune conditions. Both drugs have *in vitro* activity against SARS-CoV-2, with HCQ having relatively higher potency. HCQ can be used during breastfeeding and pregnancy even though it crosses the placenta. Because there does not appear to be fetal toxicity and breastfed infants are exposed to only 2% of the maternal dose, it is considered to be safe (41). It is well-known that chloroquine has been used for more than 20 years in regions with malaria, with no side effects either on pregnancy or the fetus (31, 42).

The drug dose necessary to treat a viral infection is lower than in malaria (31), and the majority of authors agree with the following protocol: if the patient's weight is ≥ 50 kg, 500 mg x 2/day for 7 days; if the weight is <50 kg, 500 mg x 2/day in the first 2 days, 500 mg x 1/day from the third to the 7th day (4). The contraindications and cautions for HCQ are: QT prolongation, G6PD deficiency, epilepsy, porphyria, myasthenia gravis and retinal pathology. Serious adverse events generally result from prolonged use. Complications may include cardiomyopathy, torsade des pointes, bone marrow suppression (thrombocytopaenia, agranulocytosis, and leukopaenia), hypoglycaemia. These drugs should be used with caution in diabetic patients (43).

There are insufficient data to show the benefits of HCQ or chloroquine in the treatment of COVID-19 in pregnant women, mostly given the lack of clear benefit; according to the literature, these drugs are no longer recommended for COVID-19 treatment (22). Studies around the world have highlighted the potential for the toxicity of these drugs and, in some institutions, studies were stopped because of a higher mortality rate. Therefore, the US FDA revoked authorization for these agents in patients with severe COVID-19, noting that the known and potential benefits no longer outweighed the known and potential risks (44).

## Antivirals

### Lopinavir/Ritonavir (LPV/r)

Lopinavir and ritonavir are anti-retroviral protease inhibitors that are currently approved for the treatment of HIV infection (32). LPV/r has been chosen in the treatment of coronavirus infection due to its attachment *in vitro* to SARS-CoV-1 and to the sequence similarity between SARS-CoV-1 and SARS-CoV-2 (31). This drug has been widely used during pregnancy, based on data concerning the safety and efficacy of its use in pregnant women known to be HIV-positive. No teratogenic effects or preterm labor have been observed (31, 45). After several clinical trials for the treatment of COVID-19, lopinavir 400 mg/ritonavir 100 mg for COVID-19 patients diminished the risk of adverse clinical outcomes (acute respiratory distress syndrome [ARDS] or death) (46). For adults, LPV/r are used at 200 mg/50 mg 2cp x 2/day, not exceeding 10 days of treatment and ideally in the first 7–10 days, when the peak phase of virus replication occurs (4, 31, 47).

The most common side effects are nausea, vomiting, diarrhea, abdominal pain, anorexia, gastritis, cutaneous manifestations, insomnia and anxiety. More serious adverse effects may include QT prolongation, AV block, anemia, leukopaenia, neutropaenia, hyperglycaemia, renal failure, pancreatitis and hepatotoxicity. Lopinavir/ritonavir is contraindicated in cardiac disease and liver disease (43). Moreover, it is important not to forget that 20–30% of patients with COVID infection have transaminase elevation (31).

Taking into account all the information above, LPV/r remains a choice of treatment for pregnant patients infected with coronavirus. However, lopinavir/ritonavir appear to have minimal role in the treatment of COVID-19 infection. Trials on this matter are ongoing, but it should not be neglected the fact that there are some studies that mention the possible crossing of the placenta by this drug (44, 48).

### Remdesivir

Remdesivir is a novel, investigational, intravenous drug with broad antiviral activity against SARS-CoV-2 and seems to be effective in mild to severe forms of COVID-19 infection to reduce pulmonary pathology due to its characteristic of reducing viral replication by inhibiting RNA dependent RNA polymerase (31). The recommended dose is 200 mg IV on day 1 (loading dose), followed by 100 mg IV daily, up to 10 days. The possible side effects are gastrointestinal intolerance and hepato- and renal toxicity. Several authors suggest that remdesivir should not be used in combination with other experimental antiviral agents (49). This drug has been used without fetal toxicity in pregnant women receiving supplemental oxygen, intubated or not, and in non-severe disease (45).

Several studies revealed its safety during pregnancy (31), but we find it important to mention that the literature around the efficacy of remdesivir is continually changing. If in the beginning of the COVID-19 pandemic, authors reported that this drug improved the time to recovery in patients with severe coronavirus symptoms (31), more recent trials have demonstrated that remdesivir not only does not lead to a shorter hospital stay, but also it does not minimize the risk of death (22, 50).

### Antibiotics

COVID-19 itself is not an indication for antibiotics, but regarding the possibility of a superimposed bacterial pneumonia, some protocols recommend it. The decision regarding the choice of antibiotic and initiation of antibiotic therapy should depend on the culture results of blood, urine and other fluids and on COVID-19 symptom severity. In mild COVID-19 patients, it is recommended to choose based on the patient's condition and wait for the culture results if possible, in order to administer a specific antibiotic. In severe COVID-19 patients, it is suggested to cover all possible organisms until culture results are available (31).

Azithromycin is a macrolide antibiotic which is known not only for its antimicrobial properties, but also for its immunomodulatory activity. As a consequence, macrolides are commonly used in infectious pneumonia and in inflammatory lung disease. Of all the macrolides, azithromycin is considered to have the strongest immunomodulatory effects (51). Azithromycin 500 mg (first day), followed by 250 mg every 24 h for up to 5 days, orally or intravenously, seems to be adequate for a pregnant woman (40). The most common side effects are abdominal pain, diarrhea, nausea and vomiting (31). Some of its contraindications are myasthenia gravis, torsade des pointes, prolongation of QT interval and liver failure. It is appropriate to avoid the indiscriminate use of antibiotics, especially those with a broad spectrum of action (4). Azithromycin was used in combination with HCQ for treating COVID-19 at the beginning of the pandemic, but recent studies have shown that there is no clinical benefit. Crucially, the rate of cardiac arrest is higher because of the potential adverse effects of both of these drugs (QTc prolongation) (44).

Amoxicillin is a beta lactam antibiotic and is used for most of bacterial infections as it has activity against both Gram-positive and Gram-negative bacteria. It is well-tolerated, and side effects are rare: nausea, vomiting and diarrhea. It is important to mention that as amoxicillin is a semi-synthetic penicillin, so a skin rash, erythema and anaphylaxis may appear if hypersensitivity is present (31).

It has to be taken into consideration the possibility of starting ceftriaxone 1–2 g every 24 h intravenously and teicoplanin 400 mg every 12 h for 3 doses followed by 400 mg every 24 h if the patient has alveolar infiltration and/or elevated procalcitonin (suspected bacterial superinfection) (40) until the culture results (blood, urine and/or other fluids) arrive, and after that to continue with a specific antibiotic as soon as possible (31).

All the Antibiotic Drugs Mentioned Above are Safely Used During Pregnancy and Breastfeeding (31, 52–54).

### Corticosteroids

For pregnant patients at high risk of preterm delivery within 7 days, between 24+0 and 33+6 weeks of gestation, there are clear benefits of antenatal corticosteroid administration. However, at 34+0 to 36+6 weeks of gestation, the neonatal benefits are less clear, so it is suggested to not administer corticosteroids to such patients. It is recommended to initiate therapy with the usual doses of dexamethasone (4 doses of 6 mg given intramuscularly 12 h apart) or betamethasone (2 doses of 12 mg given intramuscularly 24 h apart) in order to induce fetal pulmonary maturation. In addition, this therapy should be followed by prednisolone (40 mg orally daily) or hydrocortisone (80 mg intravenously twice daily) to complete the maternal steroid course. The objective is to avoid fetal exposure to a prolonged course of dexamethasone or betamethasone, which may have some adverse effects by crossing the placenta (long-term neurodevelopmental impairment, increased risk of preterm birth) (48).

Corticosteroids are recommended specifically for severe illness and should not be routinely used in the prevention or treatment of mild to moderate COVID-19 (44). The main adverse effects of these drugs are hyperglycaemia and hypernatraemia, but low-to-moderate doses are harmless. This is another reason why dexamethasone should be followed by prednisolone (orally) or hydrocortisone (intravenously) (55–57). It is also important to pay attention to pregnant women with gestational diabetes, pre-existing diabetes and mostly if the patient is on insulin treatment. Some studies show that betamethasone may worsen the situation, so authors suggest the administration of only one dose of betamethasone (12 mg) to keep the patient's blood sugar as normal as possible (31). Recent publications mention that methylprednisolone (1–2 mg/kg per day) should replace dexamethasone, as there is little actual data regarding the consequences on breastfeeding when dexamethasone is administered (22, 31).

### Low Molecular Weight Heparin

Direct data on thromboembolic risk with COVID-19 are limited but suggest an increased risk (58). All pregnant women admitted with COVID-19 infection or suspected COVID-19 infection should receive prophylactic low molecular weight heparin (LMWH) in a dose of 4000 IU per day unless birth is expected within 12 h (23, 31). If the pregnant woman is close to delivery, it is generally preferred to use unfractionated heparin rather than LMWH due to its readily reversible properties (58).

All pregnant women with confirmed COVID-19 infection should be prescribed at least 10 days of prophylactic LMWH (e.g., enoxaparin 40 mg daily subcutaneously) after hospital discharge. At the same time, postnatal care for women immediately following hospitalization for confirmed COVID-19 illness, which includes the birth of the baby, should undergo at least 10 days of prophylactic LMWH, regardless of the mode of birth (23).

Postpartum venous thromboembolism (VTE) prophylaxis in women with COVID-19 should be considered based on an individual risk assessment. We have noted a considerable variation in practice. For patients who did not receive antepartum prophylaxis because of COVID-19, it is not necessary to administer postpartum prophylaxis in non-severe illness and with no standard indication for postpartum VTE prophylaxis. On the other hand, for patients who received antepartum prophylaxis because of COVID-19, some studies suggest stopping treatment upon hospital discharge if there are no risk factors for VTE (e.g., recent surgery, immobilization). Nonetheless, other authors recommend continuing prophylaxis for 7 to 14 days (and up to 6 weeks) in pregnant women who had moderate/severe disease or mild disease with VTE risk factors (59–61).

There are four important concepts that will lead the medical practitioners in deciding when, how and for how long a pregnant patient with coronavirus infection will receive anticoagulation prophylaxis and/or treatment: the severity of the illness; if the woman is in hospital care or at home, in isolation; if the delivery is approaching or not; and if the patient presents any comorbidities or complications which may put her at a high risk of thrombosis (22).

### Other Therapies

It is true that, in the absence of other options, some institutions may choose to use certain agents like interleukin [IL]-6 pathway inhibitors and interferon beta. It is obvious that we need more research and clear data on the treatment of SARS-CoV-2.

Tocilizumab is an anti-inflammatory monoclonal antibody with IL-6-inhibitory effects, usually used for cytokine release syndrome and rheumatic disease. Currently, it is recommended for the treatment of critical and severe COVID-19 infection exactly due to its properties of decreasing elevated pro-inflammatory cytokine levels (e.g., IL-6) and marked elevated inflammatory markers (e.g., C-reactive protein, ferritin, D-dimer) associated with severe COVID-19 disease (22, 44). The suggested doses are 4–8 mg/kg, usually 400 mg diluted in 0.9% NaCl solution, with an infusion time of 1 h. The same dose can be re-administered after 12 h if little benefit is seen after the first administration. The maximum dose for each administration is 800 mg. Attention should be paid to allergic reactions and to contraindications, especially tuberculosis (4).

The results of the few trials on pregnant women using tocilizumab are not so reassuring. Cases of miscarriage, preterm birth and even stillbirth are mentioned. Furthermore, congenital malformations were also detected, so if the pregnancy is advancing an extra ultrasound at around 20 weeks of gestation is suggested. Currently, more studies are needed about the use of tocilizumab for COVID-19 treatment in pregnant and lactating women (31).

Interferon beta B1 is a cytokine in the interferon family with an immunomodulatory role, capable of enhancing innate and adaptive viral immunity (62). According to clinical trials, these drugs are safe during pregnancy. The risk of miscarriage, premature birth, stillbirth and fetal malformation is reported to be low. In the current literature, there are reviews that describe the interest of this treatment for COVID-19 infection, alone or in combination with other antiviral drugs, especially after finding that SARS-CoV-2 is sensitive to both interferon alpha and interferon beta (31). The interest in interferon beta is mostly in non-severe COVID-19 patients, but only in combination with ribavirin and/or lopinavir/ritonavir. Randomized trials have shown that the three drugs together are more efficient than interferon beta with lopinavir/ritonavir alone in terms of clinical improvement and hospital discharge. Also, there less time to a negative SARS-CoV-2 reverse transcription polymerase chain reaction (RT-PCR) test on a nasopharyngeal swab after triple therapy (44). Nonetheless, the WHO has recently established that treatment with interferon for COVID-19 infection does not lead to any significant improvement in the patient (22). More studies are needed to clarify the role of interferon beta in COVID-19 treatment for pregnant women (44).

### Convalescent Plasma

Convalescent plasma from patients who have recovered from COVID-19 infection has been used as a treatment for patients with severe or life-threatening COVID-19 (44), but trial data on its use are still emerging (31). Some authors have concluded that convalescent plasma is efficient in COVID-19 patients who are not severe or critically ill but in a state of immunosuppression (63). Current evidence shows that, on one hand, convalescent plasma improves the rate of nasopharyngeal viral RNA clearance (compared with standard treatment alone), but on the other hand, there is no significant difference in clinical improvement or mortality rate (44).

The donors have to be between 18 and 55 years old, with a weight > 50 kg (for men) or > 45 kg (for women), more than 2 weeks since last blood donation, and at least 2 weeks after recovery. Plasmapheresis is the collection method, with 200–400 mL collected each time. The blood samples must be tested for SARS-CoV-2 by nucleic acid testing and for SARS-CoV-2 specific IgG and IgM antibodies, in addition to general quality tests. It is generally well-tolerated at a dosage of 400 mL for one infusion or 200 mL per infusion for multiple infusions. Patients with a history of allergy to plasma, methylene blue or sodium citrate present contraindications for convalescent plasma (63).

There are a few studies in the literature with a small number of patients, but with results that should not pass unobserved. They showed good results in oxygen saturation after 3 days of plasma infusion, improvement in lung lesions after 7 days of convalescent plasma treatment and a better clinical condition of the patient (31).

For the general population, there seems to be no important side effects after receiving convalescent plasma for COVID-19 treatment (31), but currently there are data neither supporting nor refuting its use in pregnant women after a close look at recent publications. The authors consider that there is a need for more data, especially concerning the efficacy and safety of this therapy for COVID-19 pregnant or lactating patients (31).

## Discussion

In this brief review of the literature concerning the prevention and the treatment for COVID-19 infection during pregnancy and in the immediate postpartum, we intended to highlight and summarize the main drugs that provided the best results until the present moment. Pregnant patients have a higher risk of developing sever symptoms if infected with coronavirus, especially if they are more than 35 years old and and/or with a high BMI and if they present comorbidities such as diabetes, hypertension and/or cholestasis, which often occur in the second trimester of gestation (22).

The maternal adaptations to pregnancy place women in a more difficult management state if cardiopulmonary decompensation occurs. The pregnant woman brings together two patients while knowing that the priorities will be defined according to the gestational age. The pandemic emergency has led to the administration of numerous treatments without proof of effectiveness and without guarantees of no fetal effect in the long term. The fetal-maternal transmission of COVID-19 is probably low and has been demonstrated in very few clinical trials. Placental inflammation by COVID-19 followed by infection of the fetus is a possibility that we must take into account in the management of these patients (64).

In this article, we presented the drugs for potential treatment for COVID-19. Based on a comprehensive evaluation, we prioritized these possible treatments, and presented the dose-response and dose-toxicity effects for each drug. In pregnant women, it is important to adjust the treatment and to choose the timing of delivery. Above all, prevention is essential; pregnant women should follow the same recommendations as non-pregnant persons to avoid exposure to the virus (social distancing, wearing a mask in public, disinfecting surfaces, hand hygiene) (48). Regarding the newborn, there is no contraindication to breastfeeding in the case of a postpartum woman with COVID-19 infection, but precautions are recommended (surgical mask, hand, and breast hygiene) (40, 65).

Even if up to the present moment pregnant and lactating women have not been included in COVID-19 vaccination trials, experts highly recommend vaccination to avoid SARS-CoV-2 infection in patients during pregnancy and breastfeeding. The COVID-19 vaccines (either mRNA or viral vector) do not contain virus that replicates, so it is strongly suggested that pregnant and lactating women with a high risk of exposure to coronavirus or developing a severe disease if infected should undergo COVID-19 vaccination despite the non-specific side effects that may occur (11, 20). It is also important to pay attention to the type of vaccine administered, since some in a single dose while others require a two dose-series, to respect the timing between the two doses and to acknowledge that the second dose should be with the same type of vaccine as the first one (12, 19). Clear guidance is needed on the subject of COVID-19 vaccines administered during pregnancy and postpartum as, until now, this vulnerable population has been excluded from trials. Pregnant women with their obstetricians must decide to accept COVID-19 vaccination or not based on the limited data available at the moment (11, 12, 20).

Of the antiviral agents that have been evaluated, lopinavir/ritonavir appear to have minimal to little role in the treatment of SARS-CoV-2 infection, but remdesivir remains promising for the treatment of COVID-19, especially in severely ill pregnant women, as it has no reported fetal toxicity (48). It is appropriate to avoid the indiscriminate use of antibiotics, especially those with a broad spectrum of action (4). Bacterial pneumonia is seldom found during the hospital course, especially in patients who are intubated, but antibiotics may be stopped in <48 h if there is no evidence of bacterial infection (bacterial cultures and procalcitonin results) (43). At the beginning of the pandemic, azithromycin was highly used because of its antimicrobial properties and immunomodulatory activity (51), but recent studies have shown no clinical benefit (44).

The administration of antenatal corticosteroids prior to anticipated preterm birth is controversial in COVID-19 infection, but still important for patients at a high risk of preterm delivery between 24+0 and 33+6 weeks of gestation (58). Considering the studies and reviews that we analyzed on the subject of corticosteroids as a treatment for COVID-19 infection during pregnancy, we may conclude that experts confirm that the decision of corticosteroid therapy should be evaluated individually for each case.

Several studies suggest a high rate of thromboembolic complications among hospitalized patients with COVID-19, particularly those who are critically ill. Additionally, pregnant women admitted with COVID-19 infection, both suspected and confirmed, should benefit from prophylactic low molecular weight heparin (LMWH) (44, 65).

Other therapies are being used as treatment in critically patients, such as tocilizumab and interferon, but there are safety concerns regarding their use in pregnant women (40). To establish the safety of these drugs during pregnancy and postpartum for patients with COVID-19 infection, further studies are needed (31).

It is possible that convalescent plasma provides a clinical benefit in severe COVID-19 infection and also in patients who do not require mechanical intubation, but this remains uncertain for the moment, especially for pregnant women.

None of these treatments are contraindicated during pregnancy or breast-feeding, but require informed consent for use (40).

When choosing a certain drug for a patient infected with coronavirus during pregnancy or breastfeeding, physicians should take into consideration all the risks and benefits specific for each patient, knowing the lack of clear information on the safety and effectiveness of available treatment for this population (31). Taking into consideration that, at the present time, there are no clear management strategies for treating COVID-19 infection in pregnant women, it is obvious that prevention remains the principal strategy, even though we need more information about the safety and efficacy of SARS-CoV-2 vaccines. It is a certain fact that the literature about the coronavirus disease and COVID-19 vaccine for pregnant and lactating patients is evolving rapidly and that the guidelines around the world are constantly being updated and expended (11, 20). In the absence of sufficient data regarding COVID-19 in pregnant women, it is suggested to follow the same recommendations as non-pregnant persons for avoiding exposure to SARS-CoV-2, the virus that causes COVID-19, and in terms of treatment, regardless of disease severity (46).

It is important to acknowledge the speed and the rapidity of clinical trials and development of management, treatment and prevention related to COVID-19 disease during pregnancy since December 2019 until now. At the same time, the fact that study results concerning COVID-19 infection in pregnant and lactating women are coming out so fast may be a limitation of any review of the literature.

## Conclusions

The different types of treatment presented are safe during pregnancy and lactating period, with no teratogenic effects and minimal exposure to breastfed infants, but their effectiveness remains limited or even absent against the COVID-19 infection. It is important to stay alert that the pregnancy constitutes a state predisposing to thromboembolic complications exacerbated by the COVID-19 infection. For this reason, the preventive administration of a low molecular weight heparin is recommended as long as the mobility of the patient is reduced by the infection. The corticosteroids are to be taken into account for their role for the fetal pulmonary maturation between 24+0 and 33+6 weeks of gestation but also for their benefice in the management of pregnant patients with pulmonary COVID-19 involvement. Antibiotics (amoxicillin, ceftriaxone) are useful only in case of a co-bacterial infection. Therapies like tocilizumab, interferon beta B1 and convalescent plasma are used in critical and life-threatening COVID-19 infection but the data are too limited in pregnant women.

Concerning the vaccination, we strongly advise all pregnant women in the second and third trimester to receive the COVID-19 vaccine using a shared decision-making model with healthcare providers. These patients must be recorded in a comprehensive vaccine registry because additional studies are needed to examine rare adverse outcomes following vaccination during pregnancy. The COVID-19 vaccination does not represent an absolute protection again a re-infection and those cases of re-infection need to be treated with the same protocols as the no vaccinated population.

## Author Contributions

A-RE and ES conceived and drafted the original version of the article. NB developed the idea. NB and AF verified and supervised the manuscript. All authors contributed to the article and approved the submitted version.

## Conflict of Interest

The authors declare that the research was conducted in the absence of any commercial or financial relationships that could be construed as a potential conflict of interest.

## Publisher's Note

All claims expressed in this article are solely those of the authors and do not necessarily represent those of their affiliated organizations, or those of the publisher, the editors and the reviewers. Any product that may be evaluated in this article, or claim that may be made by its manufacturer, is not guaranteed or endorsed by the publisher.

## Footnotes

^1^www.ACOG.org (accessed September 16, 2020).
